# A Bibliometric Analysis of PubMed Literature on Middle East Respiratory Syndrome

**DOI:** 10.3390/ijerph13060583

**Published:** 2016-06-13

**Authors:** Zhengting Wang, Yongdi Chen, Gaofeng Cai, Zhenggang Jiang, Kui Liu, Bin Chen, Jianmin Jiang, Hua Gu

**Affiliations:** Zhejiang Provincial Center for Disease Control and Prevention, 3399 Binsheng Road, Binjiang District, Zhejiang 310051, China; ztwang@cdc.zj.cn (Z.W.); ydchen@cdc.zj.cn (Y.C.); gfcai@cdc.zj.cn (G.C.); zhgjiang@cdc.zj.cn (Z.J.); kliu@cdc.zj.cn (K.L.); bchen@cdc.zj.cn (B.C.)

**Keywords:** Middle East Respiratory Syndrome, MERS, literature review, bibliometrics

## Abstract

Middle East Respiratory Syndrome (MERS), a pandemic threat to human beings, has aroused huge concern worldwide, but no bibliometric studies have been conducted on MERS research. The aim of this study was to map research productivity on the disease based on the articles indexed in PubMed. The articles related to MERS dated from 2012 to 2015 were retrieved from PubMed. The articles were classified into three categories according to their focus. Publication outputs were assessed and frequently used terms were mapped using the VOS viewer software. A total of 443 articles were included for analysis. They were published in 162 journals, with *Journal of Virology* being the most productive (44 articles; 9.9%) and by six types of organizations, with universities being the most productive (276 articles; 62.4%).The largest proportion of the articles focused on basic medical sciences and clinical studies (47.2%) and those on prevention and control ranked third (26.2%), with those on other focuses coming in between (26.6%). The articles on prevention and control had the highest mean rank for impact factor (IF) (226.34), followed by those on basic medical sciences and clinical studies (180.23) and those on other focuses (168.03). The mean rank differences were statistically significant (*p* = 0.000). Besides, “conronavirus”, “case”, “transmission” and “detection” were found to be the most frequently used terms. The findings of this first bibliometric study on MERS suggest that the prevention and control of the disease has become a big concern and related research should be strengthened.

## 1. Introduction

Middle East Respiratory Syndrome (MERS), an emerging infectious disease, was first reported in Saudi Arabia in April 2012 and has recently spread to other Asian countries, including South Korea and China. Since April 2012 and as of 6 August 2015, 1408 cases of MERS (including 547 deaths) were reported by local health authorities worldwide, and 1064 were from Saudi Arabia [1]. The case fatality rate is around 39%. 

Bibliometrics has been utilized for evaluating scientific output and the importance of scientific studies [2]. Results from bibliometric analysis can not only provide objective information about the quantity and quality of scientific research, but also facilitate health policy decisions, allocation of the health resources and further innovative studies [3,4,5]. MERS has aroused much public concern and become a research focus for scientists throughout the world in recent years [6]. Such a concern warrants bibliometric studies on the MERS research, but none are available so far. To address such a need, we designed and performed this bibliometric study on the PubMed literature concerning MERS to map out research productivity in terms of journals, countries, organizations and individuals.

## 2. Materials and Methods

This bibliometric study analyzed MERS research articles published from 1 January 2012 to 22 July 2015. The study period was set on the assumption that all publications on MERS came after 2012, when the disease was first reported. We searched “MERS” or “Middle East Respiratory Syndrome” in “title/abstract” in PubMed and retrieved all records indexed under our predefined search strategy. The only limitation was the period of articles (2012–2015).

The retrieved articles were screened by two reviewers. Articles were included only if their primary focus was MERS. Duplicates, news reports, and book chapters were excluded. Profile information of each included article was then extracted by one of the reviewers, including the title, month and year of publication, corresponding author with his/her address, publication type and journal title, affiliation of the corresponding author, and the source country (determined according to the affiliation of the corresponding author). The data was double-checked by the second reviewer. Article retrieval and data extraction were completed within one day on 22 July 2015 to avoid possible bias resulting from the daily update of the databases. The collected data and its subtotals were used to obtain: (a) the contributions of countries, organizations and individuals to MERS research between 2013 and 2015; (b) the distribution of published papers in top journals. 

We employed VOSviewer (Center for Science and Technology Studies, Leiden University, The Netherlands) to map PubMed MERS articles’ terms. Using VOSviewer and thresholds of minimally 10 fractionally counted papers for each term, a worldwide map of the articles term was generated [7]. Each point in the map is colored in relation to the density of the items at that point. By default, this color is somewhere in between red and blue. The larger the number of items in the neighborhood of a point and the higher the weights of the neighboring items, the closer the color of the point is to red [8].

Articles were classified by two independent reviewers into three categories (prevention and control study, basic medical sciences and clinical studies, and other studies), and prevention and control studies were further divided into four research domains (transmission and risk factors, investigation and surveillance, intervention, and others) on the basis of the main objectives(s) of the study (Table 1) [9]. 

Data analysis was performed using the Statistical Package for Social Sciences (SPSS; version 19.0. IBM, New York, NY, USA) and Excel 2007 (Microsoft, Redmond, DC, USA). Impact factors (IF) for the journals were obtained in the Journal Citation Report (JCR) 2014 science edition (Thomson Reuters). We used the Kruskal Wallis Test for IF and a two-tailed probability in statistical tests, with the significant level at 0.05.

## 3. Results

**Figure 1:**
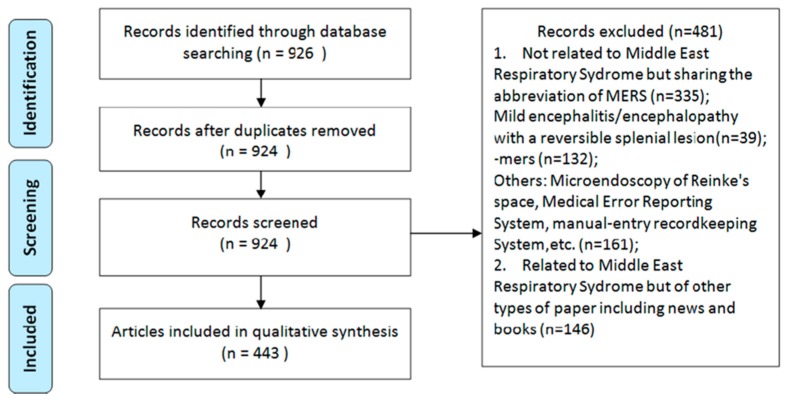
Flowchart.

A systematic search for MERS publication retrieved 926 articles in PubMed, from which two duplications were removed, leaving 924 articles. Screening through titles and abstracts excluded 481 articles (335 unrelated to MERS, 29 news, two books and 333 non-research papers). Therefore, 443 articles were included for analysis (Figure 1).

Among the 443 articles included in this study, 104 were published in 2013, 215 in 2014, and 124 in 2015. Globally, the first was published in June, 2013 and the articles published per month averaged 18. The number of monthly articles in the past 2.5 years (2013–2015) indicated a low MERS research productivity in the first few months but an obvious increase in recent months.

The retrieved articles were published in 162 journals, led by *Journal of Virology* (44 articles; 9.9%) (Table 2). The *Journal of Virology* explores the nature of the viruses of animals, archaea, bacteria, fungi, plants, and protozoa [10]. Most of the top journals are specialized in virology and infectious diseases with a high IF. Journals publishing only one article numbered 105, including *Bioinformation*, *Infection and Drug Resistance* and *Trends in Microbiology*.

The retrieved articles were from 40 countries, of which USA ranked first, followed by China and Saudi Arabia in that order (Table 3). The articles published in these three countries far outnumbered the others. Saudi Arabia is the country where MERS originated and that was most seriously stricken by the disease [1]. Fifteen countries, including Austria, Belgium and India, published only one article.

The organizations were classified into six categories, specifically government department, research organization, university, hospital, centers for disease control and prevention, and others (company, blood center, Red Cross, *etc*.). More than half (62.4%) of the research output came from universities, followed by research organizations (10%), others (9.5%), hospitals (8.5%), government departments (6.3%) and CDCs (3.2%). The three most productive organizations were the Ministry of Health of Saudi Arabia, the University of Hong Kong in China, and the University of Bonn in Germany (Table 4).

Of all the corresponding authors, Z.A. Memish, from Minister of Health for Public Health in Saudi Arabia, came first with 21 articles (Table 5) [11]. The other most productive corresponding authors are from Germany, USA, Netherlands, France and China.

Ranking of the research categories was prevention and control study (26.2%), basic medical sciences and clinical studies (47.2%), and other studies (26.6%) (Table 6). The differences between the three categories in the three years were statistically significant (*X*^2^ = 23.849, *p* = 0.000) (Table 6).

Of all the 443 articles, 378 were published in SCI journals, with a minimum IF of 0.24, maximum of 55.87 and median of 4.43, and an interquartile range between 2.85 and 6.75 (Table 7). The mean ranks of the three categories were found to be prevention and control studies (226.34), basic medical sciences and clinical studies (180.23), and others (168.03). Differences in mean ranks of IF between the three types were statistically significant (Kruskal Wallis Test *X*^2^ = 16.031, *p* = 0.000).

A total of 116 articles were identified as covering prevention and control and 99 were SCI articles with IF. The domains of the 99 SCI articles with IF were transmission and risk factors, investigation and surveillance, intervention and others (Table 8). The minimum IF was 0.59, the maximum 55.87, and the median 5.99, with an interquartile range between 3.01 and 8.88. The mean rank of IF of transmission and risk factors was 46.99, of investigation and surveillance 48.26, of intervention 53.33, and of others 56.32. The differences in the IF mean ranks between the four types were not statistically significant (*X*^2^ = 1.936, *p* = 0.586).

**Figure 2:**
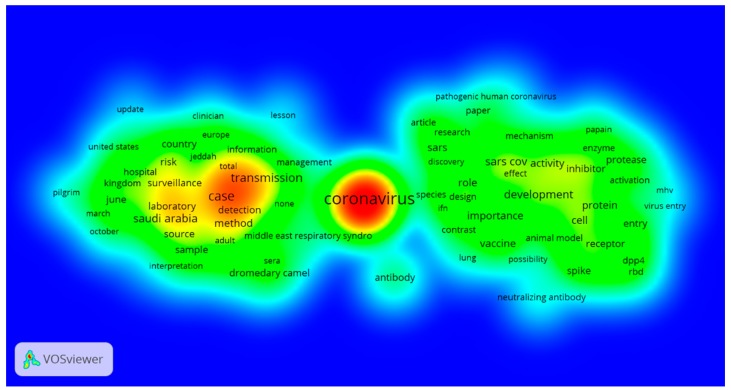
Map of frequent terms in MERS articles indexed in PubMed.

A map was created with VOSviewer, showing the density of the frequently used terms in MERS-related articles (Figure 2). Colored regions represent research areas. The font size and bubble size of a term reflect its frequency of use [7]. For example, the term “coronavirus” was more frequently used than the term “risk” because the former is in larger font than the latter. The most frequently used terms were “coronavirus”, “case”, “transmission” and “detection”. It is noticeable that less frequent terms, for example “vaccine”, had inconspicuous views.

## 4. Discussion

MERS was first reported in April 2012 and research articles on the topic have been published since 2013. Through a quick search in PubMed, we found the research outputs concerning outbreaks of other pathogens such as Severe Acute Respiratory Syndromes (SARS) and Ebola were far more than that of MERS. Since the MERS outbreak has been ongoing for more than 3 years, intuitively the science should be much more advanced. Quantity and quality of the scientific literature reflect the development and trends for a research field [12]. As far as we know, this is the first bibliometrics article to analyze the quantity and quality of MERS-based research from around the world. 

Our analysis found that most MERS research articles were published in specialized journals, of which *Journal of Virology* contained the largest number, followed by *Emerging Infectious Diseases* and *Eurosurveillance*. This may be accounted for by their contents, primarily exploring the nature of the viruses of animals, archaea, bacteria, fungi, plants, and protozoa [13,14,15,16,17,18]; by their authorship, as being chosen as target journals by the researchers in the infectious diseases area, and their high frequency of citation. 

Scientists from as many as 40 countries have engaged in research on MERS, implying that MERS has become a global public health concern and that supporting the notion that MERS research is of great significance. Among the 40 countries, the US was the most productive, followed by China and Saudi Arabia. Those top three have their own reasons for the high productivity: United States providing the largest portion of funding for the MERS research [19,20], China paying more and more attention to emerging infectious diseases, and Saudi Arabia being the origin of the disease and being the country most heavily stricken by it [21]. 

The most productive organizations were found to be the universities, which are understandably the most active and strong in scientific research. However, the most productive author was not from a university, as Z.A. Memish from the Ministry of Health for Public Health in Saudi Arabia, outnumbered all others with 21 articles, and may have served as the major source of data on MERS for other studies. This is understandable in view of his easy access to the first-hand data about the MERS outbreak in the country.

The IF of an academic journal is determined by the average number of citations of the recent articles published in that journal. Thus literature evaluations using IFs are more quantitative [22]. In contrast to their lowest proportion (26.2%) across the three years, the articles featuring prevention and control were the most frequently cited because they had higher IF than those in the other categories. At the same time, the density visualization of MERS indicated that “coronavirus” and “transmission” were two most frequently used terms. These observations suggested that prevention and control is the greatest concern in the studies. With a high case fatality rate and unavailability of an effective treatment, research on prevention and control of MERS needs to be strengthened.

Our study found that “case” and “transmission” were among the most frequently used terms, suggesting great concern of and focus on those aspects of the disease. When the articles focusing on prevention and control were further divided into transmission and risk factors, investigation and surveillance, intervention and others categories, the intervention category had the fewest articles. Since implementation of appropriate intervention can prevent transmission of MERS and reduce its cases, research on intervention needs to be strengthened.

There are several obvious limitations in our study. First, PubMed does not index all journals and we did not include journal articles included in other databases such as Scopus and Google Scholar. The results could also be biased by not exploring databases in other languages, for example Arabic, the official language of Saudi Arabia, where the most cases of MERS were reported. Second, due to the study design and time restriction, the latest articles published after August 2015 have not yet been considered in this study and this may bias the results. Third, the search terms may have missed early articles without the acronym MERS when the virus had not yet been named. However, since there are very few of those articles and most of them are descriptive studies, results could be slightly biased. Fourth, with current methodology, each article carries a single first-corresponding author, but a lot of papers involve cooperation between countries. This may underestimate the number of the articles for some countries. Fifth, the category of basic medical sciences and clinical studies can be further divided up into at least two groups, and this can bias the results to some extent.

## 5. Conclusions

To our knowledge, our study is the first bibliometric assessment of the MERS literature. We found that a large majority of MERS articles were published in highly cited specialized journals and that universities were the most productive organizations. The IFs of the articles on prevention and control were found to be the highest and the terms “coronavirus” and “transmission” were found to be the most frequently used, as shown by the density visualization. However, the proportion of the articles on prevention and control was lower than that on basic medical sciences and clinical studies and others. The findings of this study suggest that the prevention and control of MERS has become a big concern and related research should be strengthened.

## Figures and Tables

**Table 1:** Breakdown of the research domains.

Category	Research Domain	Detailed Research Objectives
Prevention and control study	Transmission and risk factors	Determine the routes of transmission, risk factors and disease determinants
	Investigation and surveillance	Describe the outbreak (time, place, and persons) Search for causative agent (identification and characteristics) Investigate transmission (determine the modes and routes of transmission, estimate the transmission probability and variability andpredict future trends of the present outbreak)
	Intervention	Describe the use of specific intervention in the population Estimate and evaluate effectiveness of the intervention Develop methods and/or tools for real-time monitoring during the outbreak
	Others	Promote planning and policy-making by health services Investigate psychobehaviors Other epidemiological studies
Basic medical sciences and clinical study		Pathogenesis Microbiology Etiology Diagnosis (development and evaluation of the sensitivity and specificity of diagnosis methods) Treatment (assessment of efficacy and adverse events) Prognosis (description of the outcomes and identification of the prognostic factors)
Others		Drugs, vaccines research and development Reviews and comments

**Table 2:** Ten most productive journals (with impact factors (IF)) with MERS articles during the period from 2012 to 2015.

Standard Competition Ranking	Journal	Total (%)	IF (2014)
1st	*Journal of Virology*	44 (9.9%)	4.439
2nd	*Emerging Infectious Diseases*	26 (5.9%)	6.751
3rd	*Euro Surveillance*	20 (4.5%)	5.722
4th	*The Lancet Infectious Diseases*	17 (3.8%)	22.433
5th	*Virus Research*	15 (3.4%)	2.324
6th	*The Journal of Infectious Diseases*	11 (2.5%)	5.997
6th	*MBIO*	11 (2.5%)	6.786
8th	*International Journal of Infectious Diseases*	10 (2.3%)	1.859
9th	*Antiviral Research*	9 (2.0%)	3.938
9th	*The Lancet*	9 (2.0%)	45.217
9th	*PLoS ONE*	9 (2.0%)	3.234
9th	*Virology*	9 (2.0%)	2.181

**Table 3:** Ten most productive countries with MERS articles during the period from 2012 to 2015.

Standard Competition Ranking	Country	Articles (%)
1st	USA	130 (29.3%)
2nd	China	64 (14.4%)
3rd	Saudi Arabia	60 (13.5%)
4th	Germany	31 (7.0%)
5th	The Netherlands	23 (5.2%)
6th	France	15 (3.4%)
7th	UK	12 (2.78%)
8th	Australia	10 (2.3%)
9th	Singapore	7 (1.6%)
10th	Japan	6 (1.4%)

**Table 4:** Ten most productive organizations with MERS articles during the period from 2012 to 2015.

Standard Competition Ranking	Organizations	Number of Documents (%)
1st	Ministry of Health, Saudi Arabia	24 (5.4%)
2nd	The University of Hong Kong, China	19 (4.3%)
3rd	University of Bonn, Germany	17 (3.8%)
4th	National Institutes of Health, USA	16 (3.6%)
5th	University of North Carolina, USA	13 (2.9%)
6th	The Erasmus University Medical Center, The Netherlands	10 (2.3%)
7th	Centers for Disease Control and Prevention, USA	9 (2.0%)
7th	New York Blood Center, USA	9 (2.0%)
8th	Loyola University Chicago, USA	8 (1.8%)
9th	The Chinese University of Hong Kong, China	7 (1.6%)
9th	University of Maryland, USA	7 (1.6%)

**Table 5:** Ten most productive corresponding authors with MERS articles during the period from 2012 to 2015.

Standard Competition Ranking	Author	Affiliation	Publication
1th	Memish, Z.A.	Ministry of Health, Saudi Arabia	21
2nd	Al-Tawfiq, J.A.	Ministry of Health, Saudi Arabia	8
2nd	Drosten, C.	University of Bonn, Germany	8
4th	Baker, S.C.	Loyola University Chicago, USA	6
4th	Reusken, C.B.	The Erasmus University Medical Center, The Netherlands	6
6th	Baric, R.S.	University of North Carolina-Chapel Hill, USA	5
6th	Jiang, S.	New York Blood Center, USA	5
8th	Gautret, P.	Aix Marseille University, France	4
8th	Hemida, M.G.	King Faisal University, Saudi Arabia	4
8th	Hui, D.S.	The Chinese University of Hong Kong, China	4
8th	Woo, P.C.	The University of Hong Kong, China	4

**Table 6:** Research categories across years (*n* = 443).

Year	Publication (*n*)	Prevention and Control Studies *n* (%)	Basic Medical Sciences and Clinical Studies *n* (%)	Others n (%)	*X*^2^	*p*
2013	104	25 (24.0)	41 (39.4)	38 (36.5)	23.849	0.000
2014	215	69 (32.1)	110 (51.2)	36 (16.7)		
2015	124	22 (17.7)	58 (46.8)	44 (35.5)		

**Table 7:** Impact factors across research categories.

Category	Publication	IF			*X*^2^	*p*
		Minimum	Maximum	Median (25%, 75%)		
Prevention and control studies	99	0.59	55.87	5.99 (3.01, 8.88)	16.031	0.000
Basic medical sciences and clinical studies	192	0.72	55.87	4.43 (3.01, 5.99)		
Others	87	0.25	45.22	4.00 (2.32, 6.26)		

**Table 8:** IF across different domains in prevention and control study (*n* = 99).

Domain	Publication	IF			*X*^2^	*p*
		Minimum	Maximum	Median (25%, 75%)		
Transmission and risk factors	17	1.36	55.87	5.99 (2.80, 7.83)	1.936	0.586
Investigation and surveillance	50	0.59	55.87	5.74 (2.92, 6.75)		
Intervention	9	0.60	45.22	6.75 (3.33, 23.00)		
Others	23	1.78	45.22	6.75 (3.93, 22.43)		
